# High performance of multiplex fluorescence in situ hybridization to simultaneous detection of BCL2 and BCL6 rearrangements: useful application in the characterization of DLBCLs

**DOI:** 10.1007/s00428-021-03084-8

**Published:** 2021-03-25

**Authors:** Federica Zito Marino, Gabriella Aquino, Matteo Brunelli, Giosuè Scognamiglio, Serena Pedron, Andrea Ronchi, Immacolata Cozzolino, Lucianna Sparano, Gerardo Botti, Luigi Panico, Anna De Chiara, Renato Franco, on behalf of the Gruppo Ematopatologia Campano

**Affiliations:** 1 Pathology Unit, Department of Mental and Physical Health and Preventive Medicine, Università degli Studi della Campania “L Vanvitelli”, Via Luciano Armanni, 5, 80138 Naples, Italy; 2 Pathology Unit, Istituto Nazionale Tumori, IRCCS Fondazione G. Pascale, Via Mariano Semmola, Naples, Italy; 3 Pathology Unit, Department of Pathology and Diagnostics, University and Hospital Trust of Verona, P.le L.A. Scuro n. 10, 37134 Verona, Italy; 4 Pathology Unit, ‘Andrea Tortora’ Hospital, Via Andrea Tortora, 84016 Pagani Salerno, Italy; 5 Pathology Unit, Monaldi Hospital, Via Leonardo Bianchi, 80131 Avellino, Italy

**Keywords:** Diffuse large B-cell lymphoma (DLBCL), *MYC* rearrangements, *BCL2* rearrangements, *BCL6* rearrangements, Multiplex fluorescence in situ hybridisation, Fluorescence in situ hybridisation multiprobe BCL2/BCL6

## Abstract

Chromosomal rearrangements involving *BCL2*, *BCL6* and *MYC* are commonly found in the most frequent B cell lymphomas, namely follicular lymphomas (FLs) and diffuse large B-cell lymphomas (DLBCLs). Particularly, *BCL2*-rearrangement represents a diagnostic hallmark in FLs, whereas *MYC* translocation can occur simultaneously with *BCL2* and/or *BCL6* rearrangements, defining a specific category of DLBCLs with a poorer prognosis. In this study, we aim to validate the diagnostic performance of multiplex BCL2/BCL6 FISH approach in formalin-fixed paraffin-embedded FLs and DBCLs and cytological samples of DLBCL comparing to the classic set of single break-apart probes. We collected a series of lymphomas, including 85 DLBCLs, 45 FLs and 36 other B-cell lymphoma histotypes and 16 cytological samples of DLBCLs. *MYC*, *BCL2* and *BCL6* rearrangements were previously assessed by a classic FISH test using single break-apart probes. All samples were analysed by a multiplex FISH assay. In the FL series, 38 cases showed *BCL2-*R; in the DLBCLs series, *MYC*-R was detected in 21 out of 85 DLBCL patients, *BCL2-*R in 10 out of 85 and *BCL6*-R in 33 out of 85. In the DLBCL cytological series, *MYC*-R was detected in 4 out of 16, *BCL2*-R in 4 out of 16 and *BCL6*-R in 1 out of 16. Notably, in FFPE, 13 double-hit lymphomas (DHLs) and 3 triple-hit lymphomas (THLs) were detected; in the cytological series, only 3 DHL cases were observed. The dual BCL2/BCL6 FISH probe test results were fully concordant with the results obtained using classic BCL2 and BCL6 single break apart. Particularly, multiplex FISH to simultaneously detect *BCL2-*R and *BCL6*-R on a single slide could find a wide application in the characterisation of double- and triple-hit DLBCLs.

## Introduction

Chromosomal rearrangements commonly occur in B-cell lymphomas, mainly involving *BCL*2 gene at 18q21 chromosomal locus, *BCL*6 at 3q27 and *MYC* at 8q24 [1]. Such translocations arise in the most common histotypes, i.e. follicular lymphomas (FLs) and diffuse large B cell lymphomas (DBCLs) [1]. Particularly, FLs, accounting for 15–30% of all lymphomas, carry *t*(14;18)*IgH/BCL2* in 80–90% of the cases. Therefore, such translocation is considered the hallmark for the diagnosis of FL [1]. In addition, other rearrangements can be found in FLs, such as chromosomal translocations involving the *BCL6*-gene in 5–15% of the cases and more rarely MYC translocations [2]. Diffuse large B-cell lymphoma (DLBCL), a heterogeneous disease at biological, molecular and clinical levels, represents the most common human lymphoma. The World Health Organisation (WHO) classification of lymphoproliferative diseases, published in 2016, refined the previous DLBLC subtypes and identified four categories: DLBCL not otherwise specified (NOS), other lymphomas of large B cells, high-grade B-cell lymphoma and B-cell lymphoma unclassifiable [1]. In the new WHO classification, the high-grade B-cell lymphoma has been redefined, including in this subgroup the entities carrying *MYC* rearrangement (*MYC*-R) associated to *BCL2* rearrangement (*BCL2*-R) and/or *BCL6* rearrangement (*BCL6*-R) named as double- and triple-hit lymphomas [1]. The double-hit lymphomas (DHLs) refer to high-grade B-cell lymphomas with MYC rearrangement and the additional rearrangement of *BCL2* or *BCL6*. DHLs account for approximately 10% of all DLBCL. In particular, MYC/BCL2 DHLs are the most frequent subtype accounting for about 65% of DHL cases, followed by MYC/BCL6 DHL accounting approximately for 34% of cases [3–5]. The triple-hit lymphomas (THLs) include high-grade B-cell lymphomas with concomitant *MYC*-R, *BCL2*-R and *BCL6*-R and account for approximately 15–20% of all high-grade B-cell lymphoma cases [6, 7]. Patients with DHL and THL have an advanced-stage disease, two or more sites of extranodal involvement and a poor performance prognosis [8]. The HGBL-DH/TH represents a molecularly defined group of very aggressive lymphomas, poorly sensitive to the classical R-CHOP chemotherapy; therefore, the correct cytogetical characterisation is critical for the selection of the appropriate treatment. The revised 2016 WHO classification strongly recommends that all DLBCL cases should undergo fluorescent *in situ* hybridisation (FISH) analysis, because of the clinical impact of DHL and THL. A practical algorithmic approach for the diagnosis of mature aggressive B-cell lymphoma proposes *MYC*-R detection first, followed by *BCL2* and *BCL6* gene analyses in *MYC* rearranged cases [9, 10]. FISH using break-apart probes is currently the gold standard to detect chromosomal rearrangement for diagnostic and prognostic purpose. Particularly, it is required for the definition of the high-grade B-cell lymphoma with *MYC* associated to *BCL2* and/or*BCL6* rearrangements.

The multiprobe FISH approach is a valuable multitarget opportunity with diagnostic, prognostic and predictive relevance, as previously reported for other tumours [11]. FISH using multiprobe BCL2/BCL6 allows simultaneous identification of *BCL2*-R and *BCL6*-R on the same slide, optimising time consumption and costs. In this study, by including lymphomas potentially carrying *BCL2*-R and/or*BCL6*-R, i.e. FLs and DBCLs, and lymphomas notoriously without such translocations, we aim to validate the diagnostic performance of the FlexISH BCL2/BCL6 DistinguISH Probe in formalin-fixed paraffin-embedded, comparing to the efficacy of the classical FISH method using single break-apart probes. In addition, we evaluate such diagnostic performance also in a series of cytological samples of DLBCL, where in some specific cases, the study of *BCL2*-R and/or*BCL6*-R can refine the diagnosis.

## Materials and methods

### Study population

#### Series of formalin-fixed paraffin-embedded FLs and DLBCLs and a control series of other lymphomas histotypes

A series of formalin-fixed paraffin-embedded (FFPE) lymphomas was selected. Particularly, in order to evaluate the performance of the multiplex FlexISH BCL2/BCL6 DistinguISH Probe, the series was enriched with some cases carrying known rearrangements of BCL2 and/or BCL6, previously detected in the clinical setting. Thus, 166 cases of lymphomas were included in the study from the Istituto Nazionale Tumori ‘Fondazione G. Pascale’-IRCCS, Naples, and from the A.O. San G. Moscati, Avellino. The series included lymphomas, potentially carrying *BCL2*-R and/or *BCL6*-R, particularly 85 DLBCLs and 45 follicular lymphoma (FL), and B-cell lymphomas, without *BCL2*-R and/or *BCL6*-R, namely 20 classical Hodgkin’s lymphomas (C-HLs), 13 mantle cell lymphomas (MCLs), 3 chronic lymphocytic leukaemia/small lymphocytic lymphomas (CLLs/SLLs).

All cases were reviewed by two experienced pathologists and used for the building of three tissue microarrays (TMAs). The TMAs were built using two cores from different areas. Tissue cylinders with a diameter of 0.6 mm were punched from morphologically representative tissue areas of each ‘donor’ tissue block and brought into one recipient paraffin block (3 × 2.5 cm) using a semi-automated tissue arrayer (Galileo TMA, Integrated Systems Engineering, Milano, Italy).

#### Series of cytological DLBCL

We retrospectively reviewed archival cell blocks (CBs) with the diagnosis of DLBCL with enough available material diagnosed from November 2016 to January 2019 at Università della Campania Luigi Vanvitelli Hospital. The availability to select representative samples was considered when the sample contained more than 100 cells for the diagnosis and also in the reevaluation on H/E stained sections before the multiplex approach. Therefore, a series of 16 cytological cases of DLBCL was collected. CBs were prepared using the Thermo Scientific™ Shandon™ Cytoblock. Briefly, the samples were fixed before beginning cytoblock preparation. After the concentration of the fixed cells by centrifugation, the cytoblock cassettes had been assembled according to the Thermo Scientific™ Shandon Cytoclip™'s protocol and processed in a standard tissue processor. Sections obtained from CBs were used for FISH analysis.

### FISH

The fluorescence in situ hybridisation (FISH) assay was carried out using the Bond FISH kit on the automated Bond system (Leica Biosystems) according to the manufacturer’s instructions. The BOND FISH Kit consists of a formamide mixture recommended to reduce non-specific hybridisation of nucleic acid probes.

All cases were previously tested for *MYC*, *BCL2* and *BCL6* rearrangements by FISH using single break-apart probes, then FISH using multiprobe BCL2/BCL6 was performed.

FISH analysis was performed on TMAs and CB sections using the commercially available probes: ZytoLight® SPEC MYC Dual Color Break-Apart Probe (ZytoVision,GmbH), ZytoLight® SPEC BCL2 Dual Color Break-Apart Probe (ZytoVision,GmbH), ZytoLight® SPEC BCL6 Dual Color Break-Apart Probe (ZytoVision,GmbH) and FlexISH® BCL2/BCL6 DistinguISH Probe (ZytoVision, GmbH). Slides were counterstained with 4′, 6-diamidine-2′- phenylindole dihydrochloride (DAPI) in anti-fade solution and examined using an automated CytoVision platform (Leica Biosystems).

#### MYC, BCL2 and BCL6 single break-apart probes FISH analysis

*MYC*-, *BCL2*- and *BCL6*- rearrangements were previously identified separately on TMAs and archival CB. FISH was performed using ZytoLight® SPEC MYC Dual Color Break-Apart Probe (ZytoVision,GmbH), ZytoLight SPEC BCL2 Dual Color Break-Apart Probe and ZytoLight SPEC BCL6 Dual Color Break-Apart Probe. In both cases, they consist of two fluorescent probes, one red and one green signal, flanking the MYC, BCL2 or BCL6 break point, respectively. A minimum of 100 cells on FFPE specimens and a minimum of 50 cells on CB specimens were considered for the interpretation. The FISH analysis was considered positive in relation to the classic break-apart pattern with one fusion signal and two separated orange and green signals observed in ≥ 15% neoplastic cells.

#### Dual BCL2/BCL6 FISH analysis

All specimens were analysed using the FlexISH® BCL2/BCL6 DistinguISH Probe (ZytoVision, GmbH) for the simultaneous detection of *BCL2* and *BCL6* rearrangements on the same slide.

**Fig. 1:**
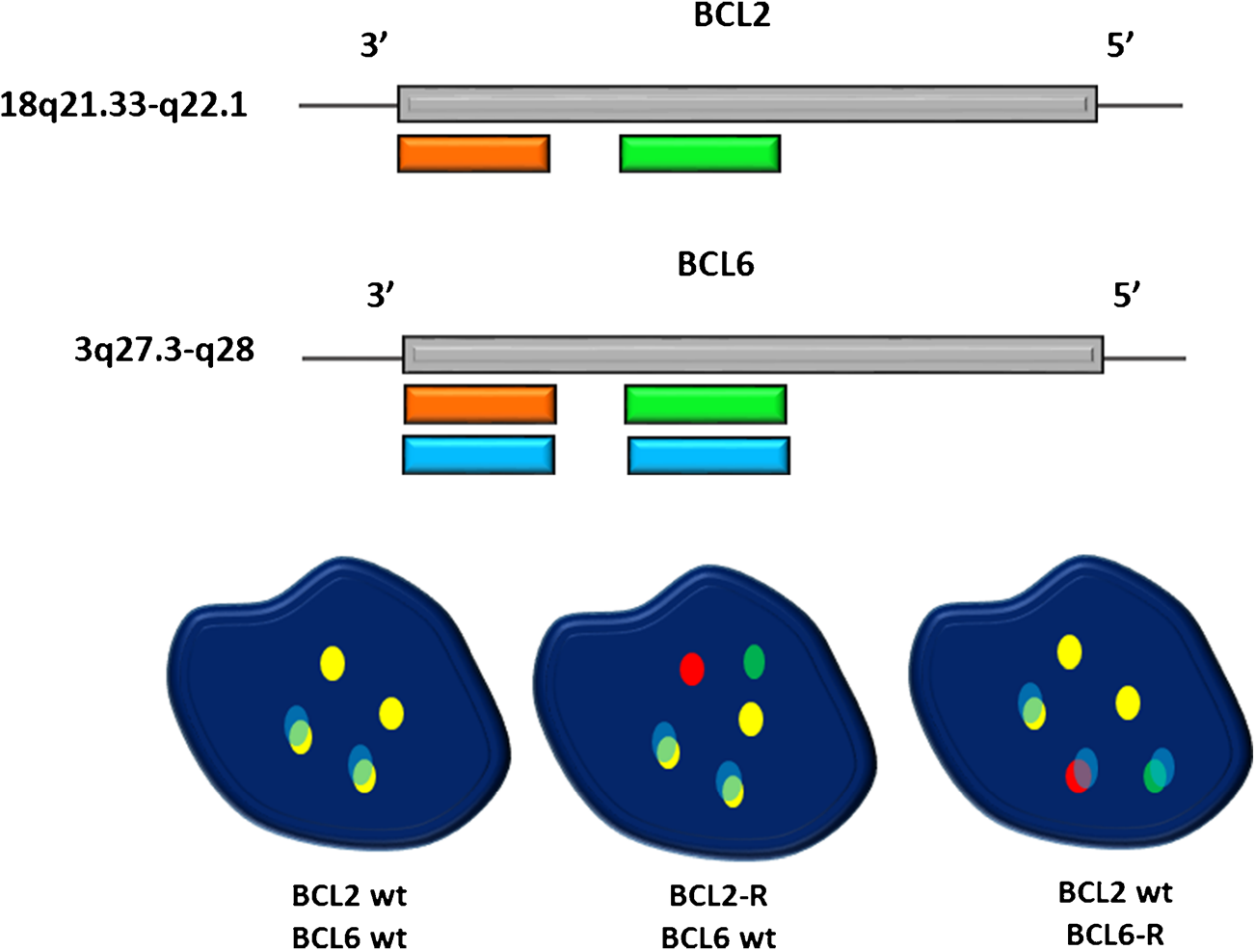
The F*lex*ISH ® BCL2/BCL6 DistinguISH™Probe’s design

The FlexISH ® BCL2/BCL6 DistinguISH™ Probe is a mixture of fluorophore-labelled DNA probes including (1) a green fluorochrome direct-labelled probe hybridises proximal to the *BCL2* breakpoint regions; (2) a green fluorochrome direct-labelled probe hybridises proximal to the *BCL6* breakpoint regions; (3) an orange fluorochrome direct-labelled probe hybridises distal to the *BCL2* breakpoint regions; (4) an orange fluorochrome direct-labelled probe hybridises distal to the *BCL6* breakpoint regions; (5) a blue fluorochrome direct-labelled probe hybridises distal to the *BCL6* breakpoint region; (6) a blue fluorochrome direct-labelled probe hybridises proximal to the *BCL6* breakpoint region. A scheme of the TheFlexISH ® BCL2/BCL6 DistinguISH™ Probe is provided in Fig. 1.

A *BCL2*-R is indicated by one fusion signal and two separated orange and green signals, both not co-localising with the blue signals. A *BCL6*-R is indicated by one fusion signal and two separated orange and green signals, all co-localising with the blue signals.

The FISH interpretation was performed with the fluorescence microscope Leica DM5500 B Automated using different filters, particularly ET-D/O/G for double SpectrumGreen plus SpectrumOrange and ET-A for the Spectrum Aqua. Briefly, the slides were first evaluated using the dual SpectrumGreen plus SpectrumOrange filter searching for split red and green signals. Signals detected with SpectrumAqua filter allowed us to distinguish between *BCL6* rearranged and *BCL2* rearranged signals, reflecting the co-localisation (*BCL6*) or absence of co-localisation (*BCL2*) of aqua signals.

## Results

### Patient characteristics

A total of 166 patients with histological diagnosis of lymphoma were included in this study. Forty-five out of 166 were FLs and 85 out of 166 DLBCLs. In the FLs series, the mean age was 65 years (range 45–89 years): 40 cases were older and 5 were younger than 60 years of age. The FLs series included 28 male patients (62%). The mean age of the DLBCL patients was 61 years (range 41–87 years): 60 cases were older and 25 were younger than 60 years of age. The DLBCL series included 47 male patients (55.2%) and 26 female patients (44.7%). In our series, 52 out of 85 DLBCLs were GC-type (61%) and 33 were non-GC-type (39%). Overall, 36 patients with other histotypes were analysed in our study: 20 C-HL, 13 MCL, 3 CLL/SLL. The mean age of patients with a histotype other than FL/DLBCL was 58 years (range, 35–88 years): 38 cases were older and 6 were younger than 60 years of age. Of the 36 patients with histotypes other than DLBCL/FL, 20 were female (55.5%) and 16 were male patients (44.5%).

A total of 16 patients with a cytological diagnosis of DLBCLs were included in this study. The mean age of DLBCL patients was 62 years (range 38–79 years): 12 cases were older and 4 were younger than 60 years of age. The series of DLBCLs included 10 male and 6 female patients. In only 2 out of 16 DLBCLs patients, we obtained histological biopsy, which confirmed the cytological diagnosis.

### MYC, BCL2 and BCL6 single break-apart probes FISH analysis

*MYC*, *BCL2* and *BCL6* rearrangements were previously assessed by the classic FISH assay using single break-apart probes.

In the FL series, 38 out of 45 cases showed *BCL2*-R, whereas *MYC*-R and *BCL6*-R were not observed.

In the DLBCLs series, *MYC*-R was detected in 21 out of 85 DLBCL patients, *BCL2-*R in 10 out of 85 and *BCL6*-R in 33 out of 85 (Table 1). Overall, 14 out of 52 GC-type DLBCLs harbouring *MYC*-R (30%), 6 out of 52 *BCL2*-R (11.57%) and 17 out of 52 *BCL6*-R (32.7%) were recorded. In our series of DLBCLs, we identified 3THLs and 13 DHLs, specifically 4 cases *MYC/BCL2* DHL and 9 cases MYC/BCL6 DHL (Table 2).

**Table 1:** BCL2 and BCL6 status in our series through classic FISH and multiplex BCL2/BCL6 FISH

	FISH single test						MULTIPLEX BCL2/BCL6					
	BCL2		BCL6		BCL2 and BCL6		BCL2		BCL6		BCL2 and BCL6	
	R	NR	R	NR	R	NR	R	NR	R	NR	R	NR
Histological samples (N. 130)												
FLs (N.45)	38 (84.4%)	7 (15.6%)	0 (0%)	0 (0%)	0 (0%)	0 (0%)	38 (84.4%)	7 (15.6%)	0 (0%)	0 (0%)	0 (0%)	0 (0%)
DLBCLs (N.85)												
MYC-wt (N.69)	3 (3.5%)	66 (77.7%)	24 (28.2%)	45 (53%)	0 (0%)	69 (81.2%)	3 (3.5%)	66 (77.7%)	24 (28.2%)	45 (53%)	0 (0%)	69 (81.2%)
MYC-R (N.16)	7 (8.2%)	9 (10.6%)	9 (10.6%)	7 (8.2%)	3 (3.5%)	13 (15.3%)	7 (8.2%)	9 (10.6%)	9 (10.6%)	7 (8.2%)	3 (3.5%)	13 (15.3%)
Cytological samples (N.16)												
DLBCLs												
MYC-wt (N.12)	1 (6.3%)	11 (68.7%)	0 (0%)	12 (75%)	0 (0%)	12 (75%)	1 (6.3%)	11 (68.7%)	0 (0%)	12 (75%)	0 (0%)	12 (75%)
MYC-R (N.4)	3 (18.7%)	1 (6.3%)	1 (6.3%)	3 (18.7%)	0 (0%)	4 (25%)	3 (18.7%)	1 (6.3%)	1 (6.3%)	3 (18.7%)	0 (0%)	4 (25%)

*R* rearranged, *NR* not rearranged, *wt* wild type, *DLBCLs* diffuse large B-cell lymphomas, *FLs* follicular lymphomas

**Table 2:** Double-hit and Triple-hit in our DLBCLs series

FFPE DLBCLs N. 85			
	DHL		THL
	BCL2-R	BCL6-R	
MYC-R	4	9	3
CB DLBCLs N. 16			
	DHL		THL
	BCL2-R	BCL6-R	
MYC-R	3	0	0

*FFPE*, formalin-fixed paraffin-embedded; *CB*, cell block; *R*, rearranged; *NR*, not rearranged; *DHL*, double-hit; *THL*, triple-hit

In the control series of other lymphomas histotypes, no cases showed *MYC*-R or *BCL6*-R or *BCL2*-R.

In the DLBCL cytological series, *MYC*-R was detected in 4 out of 16, *BCL2*-R in 4 out of 16 and *BCL6*-R in 1 out of 16 (Table 1). Thus, 3 out of 16 cytological DLBCLs were *MYC/BCL2* DHL, whilst no THLs were detected in the cytological DLBCL series (Table 2). In two out 16 cases with subsequent histological confirmation, the status of *MYC*, *BCL2* and *BCL6* status was also confirmed, being one case a DHL and the other without *MYC*, *BCL2* and *BCL6* rearrangements .

### Dual BCL2/BCL6 FISH analysis

**Fig. 2:**
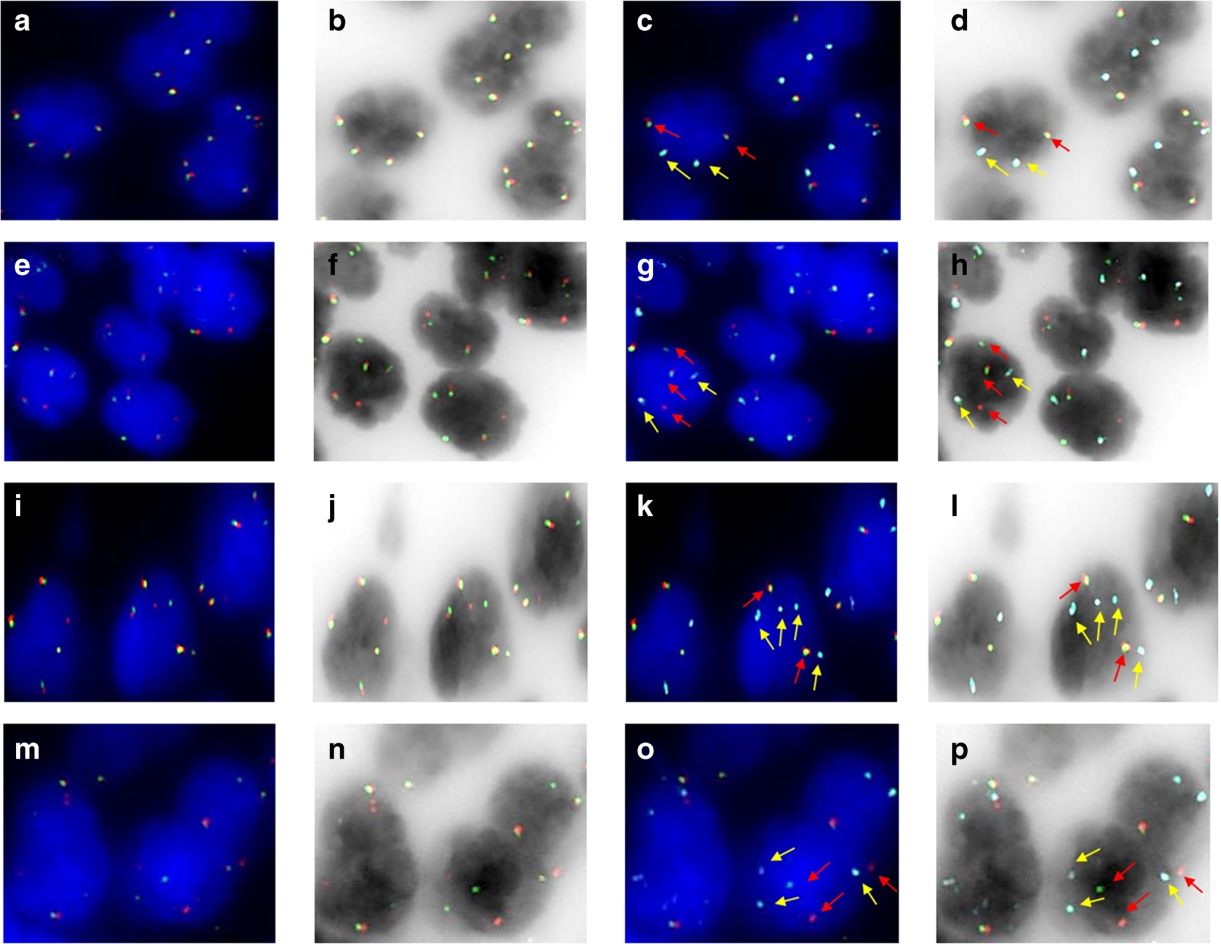
Results of FlexISH BCL2/BCL6 DistinguISH Probe FISH **a**–**d** cases with *BCL6* and *BCL2* wild type: **a**, **b** orange/green channel, **c**, **d** orange/green/aqua merged channels, red arrows indicate *BCL2* signals: 2 fusion signals, white arrows indicate *BCL6* signals: 2 fusion signals colocalised with blue. **e**–**h** Cases with *BCL2*-rearrangement and *BCL6* wild type: **e**, **f** orange/green channel, **g**, **h** orange/green/aqua merged channels, red arrows indicate *BCL2* signals: 1 fusion signal and split signals (1 orange and 1 green), white arrows indicate *BCL6* signals: 2 fusion signals colocalised with blue. **i**–**l** Cases with *BCL6*-rearrangement and *BCL2* wild type: **i**, **j** orange/green channel, **k**, **l** orange/green/aqua merged channels, red arrows indicate *BCL2* signals: 2 fusion signals, white arrows indicate *BCL6* signals: 1 fusion signal colocalised with blue and 1 green signal colocalised with blue. **m**–**p** Cases with *BCL2*-rearrangement and *BCL6*-rearrangement: **m**, **n** orange/green channel, **o**, **p** orange/green/aqua merged channels, red arrows indicate *BCL2* signals: 1 fusion signal and split signals (1 orange and 1 green), white arrows indicate *BCL6* signals: 1 fusion signal colocalised with blue and 1 green signal colocalised with blue

**Fig. 3:**
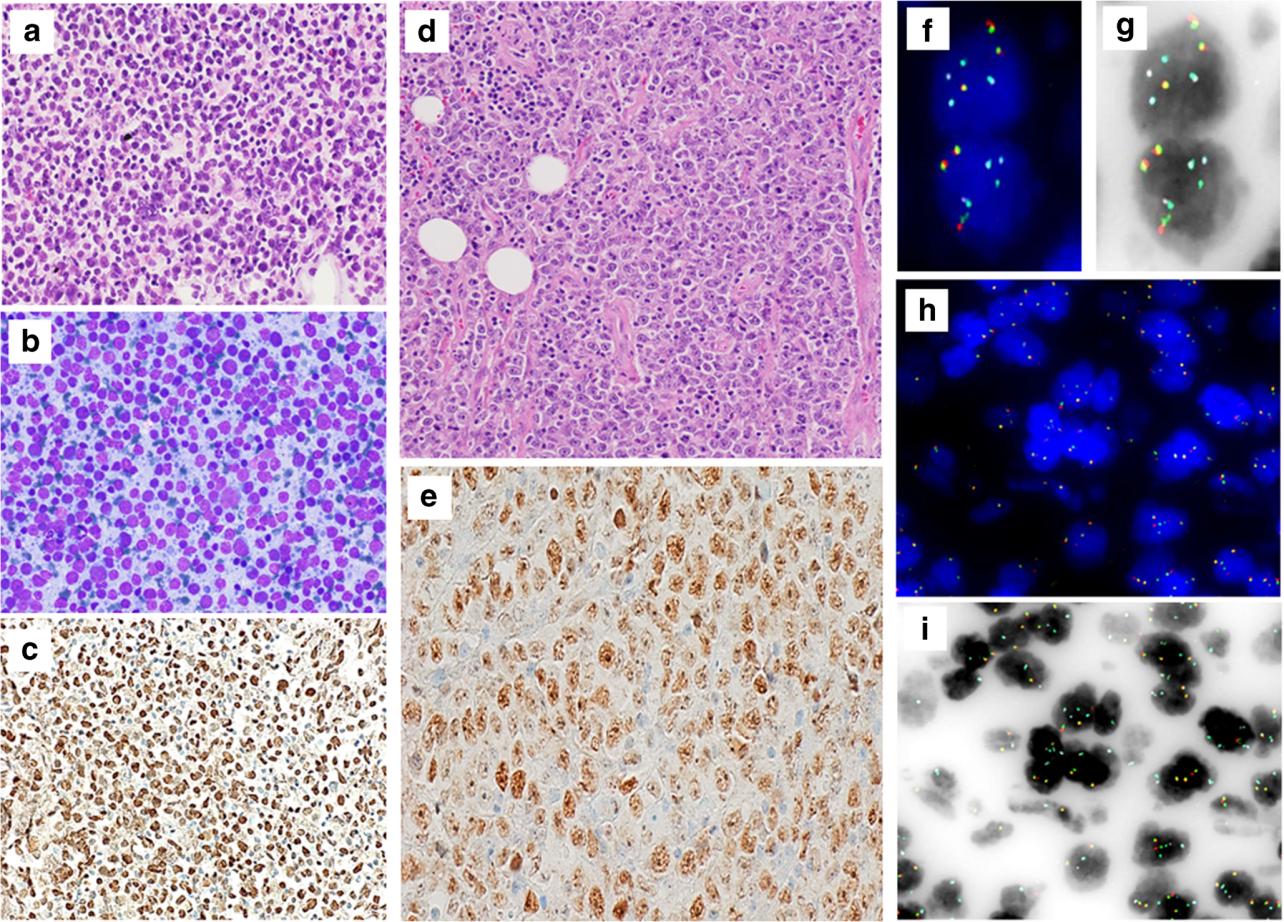
Case of diffuse large B-cell lymphoma (DLBCL) harbouring *BCL6*-rearrangement (*BCL6*-R) and *BCL2* wild type diagnosed on cytological sample and confirmed on subsequent histological sample. **a** Cytological features of DLBCLon direct smear: large isolated cells with an irregular nuclear shape and coarse, granular, dispersed chromatin. Nucleoli are evident in several cells. (Diff-Quik stain, ×40). **b** Cell-block section: large, isolated, irregular cells with dispersed chromatin (hematoxylin and eosin staining, ×40). **c** Immunostained cell-block section: diffuse, nuclear BCL6 positivity (Immunostain, ×40). **d** Histological features of diffuse large B-cell lymphoma (DLBCL) (hematoxylin and eosin staining, ×40). **e** Immunostained: diffuse, nuclear BCL6 positivity (Immunostain, ×40). **f, g** FlexISH BCL2/BCL6 DistinguISH Probe FISH (orange/green/aqua merged channels) on cell block section of DLBCL: *BCL6*-R is indicated by one fusion signal colocalised with blue, 1 green signal colocalised with blue and 1 orange signal colocalised with blue, *BCL2* wild type is indicated by two fusion signals. **h, i** FlexISH BCL2/BCL6 DistinguISH Probe FISH (orange/green/aqua merged channels) on histological sample of DLBCL: *BCL6*-R is indicated by one fusion signal colocalised with blue, 1 green signal colocalised with blue and 1 orange signal colocalised with blue, *BCL2* wild type is indicated by two fusion signals

The concordance of the results obtained from FlexISH BCL2/BCL6 DistinguISH Probe (ZytoVision, GmbH) for the simultaneous detection of *BCL2* and *BCL6* status on a same slide was evaluated in relation to the FISH results of *BCL2* and *BCL6* with single break-apart probes (2). Thus, all 48 histological cases carrying *BCL2*-R (38 FLs and 10 DLBCLs) and 33 histological cases of DLBCL carrying *BCL6*-R performed with sing probes were confirmed by the multiplex approach (Fig. 2). In addition, all cytological cases of DLBCLs carrying *BCL2*-R and/or BCL6-R documented with single probe technique were confirmed by the multiplex techniques (Fig. 3). In our series, no false-positive and false-negative results were reported using Dual BCL2/BCL6 FISH assay. The dual BCL2/BCL6 FISH assay was able to easily distinguish both classical *BCL2*-R and *BCL6*-R.

## Discussion

The diagnosis of lymphomas is challenging, as it needs to take into account morphological, immunophenotypic and molecular data, integrated with clinical features. Several lymphomas are characterised by non-random chromosomal translocations.

Thus, identifying these aberrations is required for the correct diagnosis of each specific entity. For example, identification of the BCL2/ IgH gene locus translocation in an ambiguous morphologic and immunophenotypic context may allow the diagnosis of FL, precisely because *t*(14;18) represents the hallmark of FL. However, the use of FISH represents an exception in such context, since simpler surrogate methodologies, such as immunohistochemistry, could be more easily applied.

Moreover, in some instances, the identification of specific cytogenetic alterations is required for clinically relevant subgroups. Indeed, DLBCLs with MYC rearrangement and/or MYC overexpression represent a subgroup with aggressive clinical behaviour. Furthermore, MYC alterations are frequently associated with additional genetic abnormalities. Thus, the combination of *MYC* alterations with *BCL2-* and/or *BCL6*- rearrangements led to the definition of a new diagnostic subgroup of high-grade B-cell lymphomas (HGBLs) regardless of its morphological features; thus, in this group, lymphomas previously defined as having morphology/phenotype indeterminate between DLBCL and Burkitt lymphoma were included. Therefore, a comprehensive histopathological diagnosis of HGBL should include not only IHC but also FISH. The ESMO guidelines recommend FISH testing in all patients with DLBCL to provide prognostic information for treatment purposes [10, 12].

The diagnosis workflow for DLBCLs should include *MYC*-R detection first, followed by *BCL2* and *BCL6* gene analyses exclusively in cases carrying *MYC*-R [9, 10].

FISH using break-apart probes specific for *MYC-*, *BCL2-* and *BCL6*- rearrangements are routinely used to detect DHL and THL. In this context, the multiprobe FISH approach to simultaneously detect *BCL2-*R and *BCL6-*R could provide additional information in the diagnostic algorithm of HGBL.

FISH using multiprobe BCL2/BCL6 allows simultaneous identification of *BCL2-*R and *BCL6-*R on the same slide, optimising time consumption and costs. Moreover, the use of the multiprobe BCL2/BCL6 FISH would also provide cytogenetic information related to the status of two genes rather than one in a single step, improving the diagnostic procedure.

As previously observed with ALK/ROS1 multiprobe in lung specimens, FlexISH BCL2/BCL6 DistinguISH Probe represents an easily interpretable method [11]. The interpretation of BCL2/BCL6 multiprobe FISH is not closely linked to automatic scanning and should be performed in two steps, according to the approach we previously proposed [11].

**Fig. 4:**
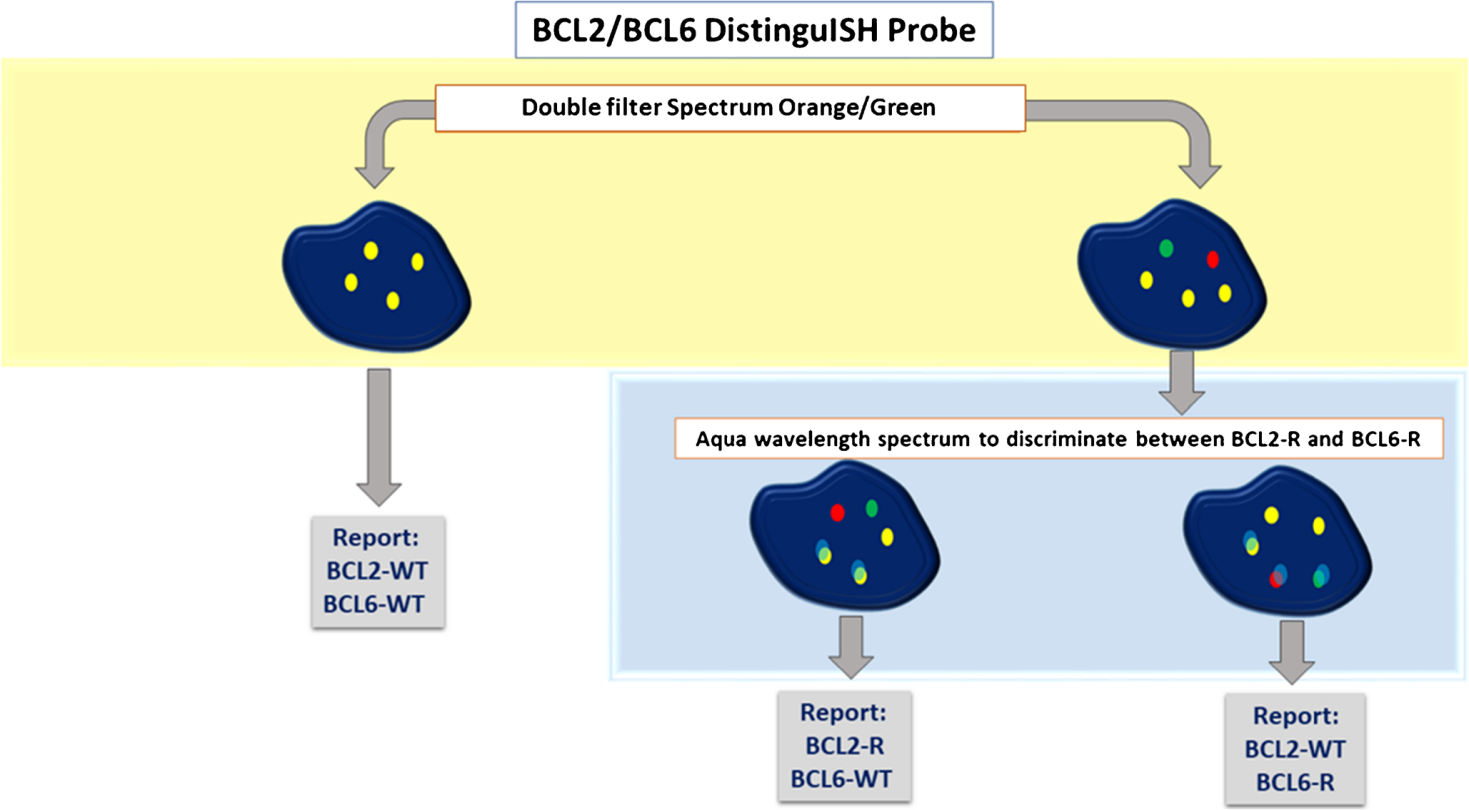
Algorithm of BCL2/BCL6 DistinguISH Probe FISH interpretation

First, the assessment of the *BCL2* and *BCL6* gene status should be performed and if the rearrangement of one of the two genes is present, the second step should discriminate between *BCL2*-R and *BCL6*-R (Fig. 4).

In our series, the FlexISH BCL2/BCL6 DistinguISH Probe showed high analytical performance in the detection of the atypical FISH patterns as well. We found no false-positive or false-negative results using the dual probe compared to the results obtained through single *BCL2* and *BCL6* FISH assays performed using the single break-apart probes for each gene. Our data demonstrated that the FlexISH ALK/ROS1 DistinguISH Probe represents a useful tool to detect simultaneously *BCL2*-R and *BCL6*-R, in FFPE and cytological samples.

Such method may be suitable for small biopsy and cytological samples, where the scarcity of the biomaterial could affect the accuracy of lymphoma diagnosis, requiring several ancillary methods. Although there has been limited use of cytology to fully characterise lymphomas, previous data have highlighted the crucial role of *MYC/BCL2/BCL6* FISH as an ancillary method to support the cytomorphologic assessments in the diagnosis of the high-grade B-cell lymphomas with clinically aggressive presentations [13–16].

The WHO recommends that all cytological samples of diffuse large B-cell lymphomas should be investigated through cytogenetic approach, since DHL and THL may appear cytomorphologically indistinguishable from other large B-cell lymphomas. Characterisation of genetic abnormalities, such as *MYC*-R, *BCL2*-R, *BCL6-*R, is often particularly helpful in the cytological diagnosis of the lymphomas. Multiprobe BCL2/BCL6 FISH could be useful in many cytological cases, particularly those limited by the small size of the sample. Notably, in some cases, the patients cannot undergo histological lymph node sampling, so the cytological sample represents the only option for the correct diagnosis of lymphoma [13–16].

The application of a multitarget BCL2/BCL6 FISH in lymphomas could improve the molecular diagnosis in terms both of time and costs. Since the cost of a test to determine a single target gene is approximately €100, the multiplex approach would reduce the cost giving the result of not one but of two target genes.

To the best of our knowledge, until now, no study has analysed the performance of multiprobe BCL2/BCL6 FISH compared to the classical FISH assay to detect the status of two oncogenes separately. Multiprobe BCL2/BCL6 FISH allows simultaneous detection of *BCL2*-R and *BCL6*-R and, additionally, discrimination between possible aberrations affecting these chromosomal regions, individually. Particularly, in our opinion, the multiplex BCL2/BCL6 FISH assay could optimise time and resources by ensuring the optimal management for DLBCL patients in daily practice.
